# Evaluation and treatment of compulsive sexual behavior: current limitations and potential strategies

**DOI:** 10.3389/fpsyt.2025.1621136

**Published:** 2025-07-03

**Authors:** Lu Zhu, Wenwen Ma, Rongqiu Zhang, Chao Wang, Bing Song, Yunxia Cao, Guanjian Li

**Affiliations:** ^1^ Reproductive Medicine Center, The First Affiliated Hospital of Anhui Medical University, Hefei, Anhui, China; ^2^ National Health Commission Key Laboratory of Study on Abnormal Gametes and Reproductive Tract, Hefei, Anhui, China; ^3^ Key Laboratory of Population Health Across Life Cycle, Ministry of Education of the People’s Republic of China, Hefei, Anhui, China; ^4^ Reproductive Medicine Center, The Affiliated Jinyang Hospital of Guizhou Medical University, The Second People’s Hospital of Guiyang, Guiyang, China

**Keywords:** compulsive sexual behavior, sexual addiction, addictive behaviors, compulsivity, sexual function

## Abstract

**Aim:**

Despite having been introduced into ICD-11, the appropriate classification and symptomatology of compulsive sexual behavior disorder (CSBD) remain controversial.

**Methods:**

In this review, we examined the historical background, epidemiological status quo, comorbidities, neuroscience theories, current diagnoses, and treatment recommendations for CSBD. Additionally, we emphasized the limitations of the current research and the prospects for future work.

**Results:**

Psychotherapy and cognitive-behavioral therapy are the preferred treatment methods for CSBD. Selective serotonin reuptake inhibitors and naltrexone are commonly used as “off-label” drugs. The diagnosis and treatment of patients with CSBD should integrate biological, psychological, and social factors with expertise in sexual medicine by employing a comprehensive and holistic therapeutic approach. This treatment aims not only to control abnormal sexual desires and behaviors but also to assist patients in achieving a healthy and satisfying sexual life and well-being.

**Conclusion:**

Future research should focus on understanding etiology, improving study population representation, correcting methodological flaws in treatment evaluation, enhancing clinician training in sexual medicine, and addressing patients’ addictions and sexual function issues. Narrowing these research gaps is crucial for improving clinical diagnosis and treatment levels and formulating targeted social intervention measures.

## 1 Introduction

For over four decades, the etiology and biological underpinnings of uncontrollable sexual behavior have been the subject of intense academic debate, continuing to this day (1). The World Health Organization (WHO) recently recognized the diagnosis of compulsive sexual behavior disorder (CSBD) in the 11th Revision of the International Classification of Diseases (ICD-11, https://icd.who.int/).

In the ICD-11, CSBD is characterized by a persistent pattern of failure to control intense, repetitive sexual impulses or urges, leading to repetitive sexual behaviors. These behaviors persist for six months or more and cause significant distress or impairment in critical areas of psychosocial functioning. The complete diagnostic criteria for this disorder are listed in **Table 1**. The establishment and discussion of diagnostic criteria for CSBD have advanced research into developing etiological models and refining treatment approaches. The inclusion of CSBD in the ICD-11 resulted in a significant impact on clinical practice and research.

**Table 1:** Diagnostic criteria for CSBD in ICD-11.

Essential (required) features for CSBD
1. A persistent pattern of failure to control intense, repetitive sexual impulses or urges resulting in repetitive sexual behavior, must be manifested in one or more of the following: a) Engaging in repetitive sexual activities has become a central focus of the person’s life to the point of neglecting health and personal care or other interests, activities, and responsibilities (yes/no). b) The person has made numerous unsuccessful efforts to control or significantly reduce repetitive sexual behavior (yes/no). c) The person continues to engage in repetitive sexual behavior despite adverse consequences (eg, repeated relationship disruption, occupational consequences, negative impact on health) (yes/no). d) The person continues to engage in repetitive sexual behavior even when the individual derives little or no satisfaction from it (yes/no).
2. The pattern of failure to control intense, sexual impulses or urges and resulting repetitive sexual behavior is manifested over an extended period (eg, 6 months or more) (Must be met)
3. The pattern of repetitive sexual behavior causes marked distress or significant impairment in personal, family, social, educational, occupational, or other important areas of functioning (Must be met). Note for rule out. Distress that is entirely related to moral judgments and disapproval about sexual impulses, urges, or behaviors is not enough to meet this requirement.

CSBD, compulsive sexual behavior disorder; ICD-11, the 11th revision of the International Classification of Diseases.

This review provides an overview of the current knowledge status of CSBD, with an emphasis on understanding the limitations of the current research and prospects for future work. In this review, compulsive sexual behavior (CSB), defined as challenges in controlling inappropriate or excessive sexual fantasies, urges/cravings, or behaviors that cause subjective distress or impair daily functioning, will be explored, along with its potential correlations with gambling and substance addictions (2). In the context of CSB, intense and repetitive sexual fantasies, urges/cravings, or behaviors may escalate over time and have been associated with health, psychosocial, and interpersonal impairments.

For this narrative review, we conducted a comprehensive literature search using databases including PubMed, Google Scholar, and Web of Science. The search timeframe was set from the inception of relevant research up to January 2025, with a focus on studies published in English. We employed keywords such as “compulsive sexual behavior”, “compulsive sexual behavior disorder”, “sexual addiction”, “hypersexuality”, and “CSBD treatment”. Although this review follows a narrative approach rather than a formal systematic review framework, we adopted principles of critical appraisal to select high-quality studies, including original research articles, review papers, and clinical guidelines. All titles and abstracts were reviewed for inclusion, followed by full paper review for studies that appeared to meet criteria. No specific scoping review model was strictly applied; instead, we prioritized seminal works, recent advancements, and studies addressing the review’s objectives on limitations and future directions.

## 2 Historical development

Research into excessive desire behaviors, including excessive craving for sex, began to emerge in the early 20th century. Early psychoanalysts observed and commented on cases where individuals developed excessive sexual desire (3). In the United States, the concept of sexual addiction first emerged in the context of CSB management and became deeply entrenched in the self-help methodologies of 12-step groups (4). This program, originally established by Bill Wilson in the 1930s to address alcohol misuse, eventually expanded to cover various addictions, leading to the creation of Sex Addicts Anonymous in 1977. In 1983, Patrick Carnes published “*Out of the Shadows: Understanding Sexual Addiction*,” introducing the concept of sexual behavior addiction to a broad clinical audience. This seminal work, grounded in clinical case reports and theoretical conjectures, rather than empirical evidence, has faced significant criticism (5). Nevertheless, research on excessive, addictive, or compulsive sexual behaviors continued to grow throughout the 1980s and 1990s, characterized by case reports, theoretical speculations, and narrative descriptions (6).

Efforts have been made in the field of psychiatry to establish diagnostic criteria related to sexual addiction. Psychiatrist Ariel Goodman proposed diagnostic criteria for sexual addiction based on the current diagnostic standards for substance abuse disorders, such as tolerance, withdrawal, and disruption of social and occupational functions (**Table 2**) (7). Carnes and his colleagues developed several self-report screening tools, such as the 25-item Sexual Addiction Screening Test and a brief screening test known as “PATHOS” (8). In 2006, Mick and Hollander introduced the term “impulsive-compulsive sexual behavior” to describe patients who exhibit impulsive traits at the onset of their behavior and compulsive traits when dysfunctional behavior persists (9). The growing attention to these empirical studies eventually led to the proposal of “Hypersexual Disorder” as a potential diagnosis in the Diagnostic and Statistical Manual of Mental Disorders (DSM-5), emphasizing the possibility of some individuals exhibiting uncontrollable sexual behavior with symptom patterns very similar to those seen in gambling and substance use disorders (**Table 3**) (10).

**Table 2:** Proposed diagnostic criteria for sexual addiction.

Definition	A maladaptive pattern of sexual behavior that causes clinically significant impairment or distress, as evidenced by three (or more) of the following criteria, all occurring within the same 12 - month period:
1. Tolerance (as defined by either of the following):	a. A need for significantly increased amounts or intensity of the sexual behavior to achieve the desired effect. b. Markedly diminished effect with continued engagement in the same sexual behavior.
2. Withdrawal (as manifested by either of the following):	a. A characteristic psychological withdrawal syndrome, physiologically identifiable changes, and/or a characteristic psychophysiological reaction upon discontinuing the sexual behavior. b. Engaging in the same (or a closely related) sexual behavior to relieve or avoid withdrawal symptoms.
3. Exceeding Intentions	The sexual behavior is often engaged in over a longer period, in greater quantity, or at a higher level of intensity than intended.
4. Unsuccessful Control Efforts	There is a persistent desire for, or repeated unsuccessful efforts to, cut down or control the sexual behavior.
5. Time-Consuming Behaviors	A great deal of time is spent on activities necessary to prepare for, engage in, or recover from the sexual behavior.
6. Neglected Responsibilities	Important social, occupational, or recreational activities are given up or reduced because of the sexual behavior.
7. Continuation Despite Harm	The sexual behavior continues despite knowledge of having a persistent or recurrent physical or psychological problem that is likely to have been caused or exacerbated by the behavior.

Data proposed by A. Goodman (7).

**Table 3:** Hypersexual Disorder Diagnostic Criteria (The diagnosis of Hypersexual Disorder should meet both criteria B, C, and D, and at least four of the five criteria listed under subcategory A).

Criterion		Description
A		Over a period of at least six consecutive months, recurrent and intense sexual fantasies, sexual urges, or sexual behaviors, occurring in association with four or more of the following five criteria:
	A1	Repetitively engaging in sexual fantasies and urges, as well as planning for and engaging in sexual behavior.
	A2	Excessive time consumed by these sexual fantasies, urges, and behavior in response to dysphoric mood states (e.g., anxiety, depression, boredom, irritability).
	A3	Repetitively engaging in sexual fantasies, urges, or behaviors in response to stressful life events.
	A4	Repetitive but unsuccessful efforts to control or significantly reduce these sexual fantasies, urges, and behavior.
	A5	Repetitively engaging in sexual behaviors while disregarding the risk of physical or emotional harm to oneself or others.
B		These sexual fantasies, urges, and behaviors, in terms of their frequency and intensity, cause significant distress or impairment in social, occupational, or other important areas of functioning.
C		These sexual fantasies, urges, and behavior are not attributable to the direct physiological effects of exogenous substances (e.g., drugs of abuse, medications) or to manic episodes.
D		The person is at least 18 years of age.
Specify		Specify the type(s) of behavior: Masturbation, pornography, sexual activity with consenting adults, cybersex, telephone sex, strip clubs. Specify if in remission: If there is no distress, impairment, or recurrent behavior, note whether this occurs in an uncontrolled environment. Also, specify the duration of remission (in months) if in a controlled environment.

Proposed by Kafka (10).

In light of new evidence from extensive research on excessive and uncontrollable sexual behaviors, the WHO’s Working Group on Impulse Control Disorders suggested the inclusion of the new diagnosis CSBD in the ICD-11 in 2018 (11). After considerable deliberation and public commentary, this disorder was ultimately included in the ICD-11 as an impulse control disorder (12). Although the diagnosis of CSBD is new, it essentially represents a reclassification of an old phenomenon. Excessive sexual behavior, hypersexuality, CSBD, and sexual addiction are different terms for the phenomenon of excessive sexual behavior, reflecting various theoretical frameworks for understanding this behavior (10, 13).

## 3 The epidemiology of CSBD

Previous epidemiological data on CSBD were mostly fragmented and criticized for methodological limitations, including non-representative samples and questionable diagnostic indicators (14). The incidence rates of CSBD vary significantly (15). In Western developed countries, 8–13% of men and 5–7% of women meet the diagnostic criteria for CSBD (16).

A recent large study involving 4,633 individuals from the general German population reported a lifetime CSBD prevalence of 4.9% in men and 3.0% in women (17). In the largest study to date that defined and operationalized CSBD according to the ICD-11 guidelines, researchers used data from the International Sex Survey (n=82,243) to validate the original version (CSBD-19) and the short version (CSBD-7) of the Compulsive Sexual Behavior Disorder Scale and compared CSBD risks across 42 countries, three genders, and eight sexual orientations (18). The study found that the global incidence rate of CSBD is approximately 5%. Country- and gender-based differences in CSBD levels were observed, but no differences were found based on sexual orientation. These variations may reflect cultural attitudes toward sexuality. For example, a study in Germany reported a lifetime prevalence of 4.9% in men and 3.0% in women, while region-specific data suggest that societies with stricter sexual morality may underreport CSBD due to stigma, or overreport it due to cultural guilt (17, 18). In addition, only 14% individuals with CSBD sought treatment.

These epidemiological findings may serve as a basis for promoting research on the prevention and intervention strategies for CSBD. Considering the compromised sexual health and well-being of individuals with CSBD, this is a serious public health issue given its current known prevalence.

## 4 The comorbidities of CSBD

The comorbidity rate between CSBD and other mental disorders is quite high. Mood disorders, particularly depression, are the most commonly associated conditions with CSBD (19). Anxiety disorders, particularly generalized anxiety disorder and social anxiety disorder, also frequently coexist with CSBD (20). Additionally, Wery et al. reported a high suicide risk among individuals with CSBD. Several studies reported that the most common personality types associated with CSBD are histrionic, paranoid, avoidant, obsessive-compulsive, narcissistic, and passive-aggressive (21, 22).

The association between bipolar disorder and CSBD is complex. In clinical practice, CSB frequently occurs alongside mania and hypomania (23). A meta-analysis of children and adolescents with bipolar disorder revealed that approximately 31–45% of adolescents experience CSB during manic episodes (24). A recent meta-analysis discovered that CSB emerged as a prodromal symptom before the first manic episode in 17% of patients with bipolar disorder, and is one of the most common symptoms before recurrent bipolar mood disorders (25). Some studies suggest that patients with CSB may exhibit more symptoms of attention deficit/hyperactivity disorders (ADHD). However, these symptoms are not severe enough to be classified as a formal diagnosis of ADHD (26).

Previous studies have also linked compulsivity to CSB (27). Compulsivity may also reflect habitual behaviors with little or no pleasure, as reflected in the CSBD criteria. Bothe et al. found a small but significant association between compulsivity and CSB in a sample of 13,778 men and women from the general population (28). However, a recent study of 539 outpatients with current impulse control disorders (OCD) (51.8% were females) found that only 5.6% had been diagnosed with CSBD and 3.3% had current CSBD. This suggests that the prevalence of CSB among patients with OCD may be comparable to that in the general population (29). Despite the use of the term compulsivity in the diagnostic category, CSBD is not considered a subtype of OCD. In contrast to CSBD, compulsive behaviors in OCD are usually a response to intrusive, unwanted, and anxiety-provoking thoughts, and are not considered pleasant.

CSB often co-occurs with behavioral addictions. In a study in Brazil that included 458 patients with gambling disorders, 6.4% reported CSB that met the criteria for mental disorders (30). Grant et al. reported that 19.6% of 225 gamblers exhibited CSBs, suggesting that similar physiological and psychological processes are associated with these two addictive behaviors. In 70.5% of patients with comorbidities, compulsive sexual behavior preceded pathological gambling, suggesting a shared mechanism of brain dysfunction (31).

CSB also frequently coexists with substance use disorders (SUDs). More than 40% of patients with CSBs are diagnosed with additional SUDs, with alcohol use disorder being the most common. Additionally, cocaine abuse was reported to be more prevalent among men with CSB than among those with paraphilias (32). Furthermore, the use of drugs such as methamphetamine, gamma-hydroxybutyric acid, and alkyl nitrates to facilitate participation in sexual activities or enhance their pleasure, described as “chemsex,” may also lead to out-of-control and dangerous sexual behaviors (33).

**Figure 1:**
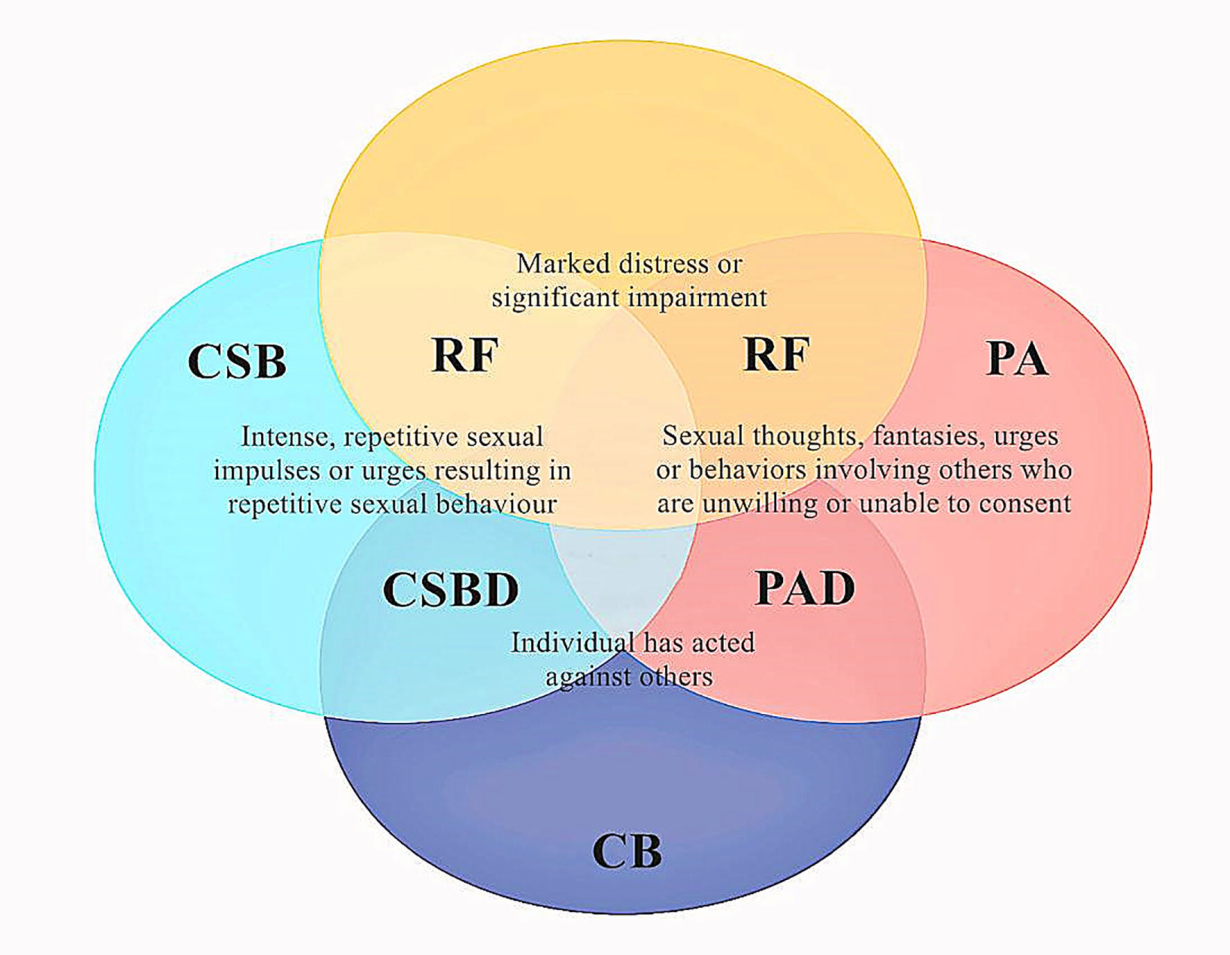
The link between paraphilia and compulsive sexuality and their association with sexual delinquency and distress. Psychological distress is a core disorder criterion for both CSBD and paraphilic disorders. CB, criminal behavior; PA, paraphilia; PAD, paraphilic disorder; RF, risk factor for sexual reoffending.

Paraphilic disorders represent another diagnostic entity that partially overlaps with CSBD. CSBD is characterized by normal sexual fantasies, whereas paraphilia is characterized by paraphilic fantasies, sexual urges, and sexual behaviors (34). The term “paraphilia” is limited to sexual behavior that involves patterns of sexual arousal directed primarily at non-consenting others or that is associated with substantial distress or an immediate risk of harm or death (35) (**Figure 1**). One diagnosis does not necessarily exclude the other, as they may exist as comorbid conditions in up to 30% of the cases (32, 36). According to the ICD-11, CSBD will not be diagnosed if an individual can exert some degree of control over their arousal patterns.

In conclusion, CSB often co-occurs with depression, anxiety, specific personality disorders, impulse control, addiction, and substance abuse. CSB may also appear as a symptom of other mental disorders, complicating the differential diagnosis in some patients. In such cases, the pattern of substance use and its relationship with sexual behavior (motivation, sequencing, and impact) should be carefully investigated. Differential diagnosis is crucial because whether CSBD is classified as a distinct medical condition or as part of another mental health disorder will influence the treatment methods. It is worth noting that CSB is also often accompanied by negative emotional states, interpersonal problems, financial difficulties, or unemployment, which in turn may have a negative impact on the symptoms of co-occurring emotional or anxiety disorders (37, 38). This may constitute a vicious cycle, leading to the persistence of CSB symptoms and co-occurring diseases.

## 5 The neurobiology of CSBD

The neurobiological mechanisms underlying CSBD and their differentials are important for determining the classification of CSBD.

Sexual pleasure is associated with the neural reward system, such as the mesolimbic dopamine pathway (39). According to the “salience incentive theory of addiction” (40), this neural activity is more about craving (“wanting”) for hedonic stimuli rather than the enjoyment or pleasure (“liking”) itself. This theory suggests that the pathological activation of the dopamine-”wanting” system underlies addictive behaviors. Neuroimaging studies indicate that patients with CSBD exhibit heightened reactivity in the limbic (wanting) region of the midbrain, similar to substance addiction. For instance, Voon et al. demonstrated that sexual cues led to greater corticostriatal activity among patients with CSBD than among healthy control participants (41). Specifically, enhanced activity was detected in the dorsal anterior cingulate cortex, ventral striatum, and amygdala. This pathway implies more “wanting” (as opposed to “liking”) arousal by patients with CSBD in response to sexual cues. Gola et al. revealed that, compared to control participants, individuals with problematic pornography use displayed greater neural activation in response to cues predicting sexual stimuli (42). This disparity was not observed when reacting to actual sexual stimuli, suggesting differences in “wanting” rather than in “liking.” These findings support the CSBD model of addiction. Klucken et al. reported slightly different results: patients with CSBD exhibited greater activity in the amygdala in response to conditioned stimuli than did the controls. However, no differences in the ventral striatum were identified between CSBD and control participants (43).

In addition to variations in cue activation between CSBD and control participants, differences in brain structure have also been noted. Volumetric differences in the striatum have been linked to SUDs, but findings are mixed, with some studies reporting decreased gray matter volume and others reporting an increase. Seok et al. also detected gray matter enlargement in the right cerebellar tonsil among patients with CSBD and those with OCD (44). Schmidt et al. found an increased volume in the amygdala, which is associated with motivational salience and emotional processing (45). They observed different results in alcohol addiction and suggested that this discrepancy might be attributed to alcohol neurotoxicity.

Diffusion Tensor Imaging (DTI) is an MRI technique that examines the integrity of the white matter by measuring the self-diffusion of water in the brain tissue. Miner et al. found no differences in DTI measures between patients with CSBD and control participants (46). However, some differences were observed in the superior frontal region, resembling patterns seen in patients with OCD, which may also imply that CSBD aligns more closely with the OCD model.

A more recent study examined men with CSBD (n=26), gambling disorder (n=26), alcohol use disorder (n=21), and healthy controls (n=21) (47). The affected individuals, as a group, exhibited smaller frontal pole volumes in the orbitofrontal cortex, though these differences were less prominent in the CSBD group. An inverse relationship was observed between CSBD symptom severity and gray matter volumes in the anterior cingulate. According to DTI data, individuals with CSBD (36 heterosexual men) exhibited significantly reduced fractional anisotropy of the superior corona radiata tract, internal capsule tract, cerebellar tract, and white matter of the occipital gyrus than did the controls (31 matched healthy individuals) (48). These regions have also been identified in OCD and addiction, indicating similar abnormal patterns in the three conditions. However, whether CSBD is closer to addiction or OCD requires further investigation.

In conclusion, since the etiological research on CSBD conducted to date is limited, the findings should be interpreted with caution. Future studies, particularly those using larger samples and adopting longitudinal designs, are necessary.

## 6 The typology of CSBD

From a research perspective, diseases classified into the same category may provide a theoretical framework for similar etiological mechanisms, contributing to the advancement of disease research. From a practical viewpoint, appropriate classification might help in the assessment of comorbid diseases and facilitate the development of new or the application of existing and proven effective intervention measures for CSBD (for instance, if CSBD is classified as an addiction, then intervention measures for addiction might also treat CSBD) (49).

Patients with CSBD experience perceptions of uncontrollable behavior, extreme guilt related to sexual gratification, and distress related to adverse consequences or a lack of satisfaction from repetitive behaviors. This may encompass feelings of guilt and shame about masturbation, the use of online pornography, intrusive sexual thoughts, and engaging in sexual activities beyond established relational or societal boundaries. CSBD is commonly referred to in the media as “sex addiction” or “pornography addiction.” Some scientific publications attribute it to the sensitization of brain dopamine functions, similar to what is observed in substance addictions (50).

CSBD is regarded as an impulse control disorder in ICD-11. However, not all researchers agree on the rationale of this classification. Many scholars argue that CSBD can also be conceptualized as an addiction (15, 51), while others suggest that its manifestations might result from cultural differences or may even be non-pathological (52–54). The Diagnostic and Statistical Manual of Mental Disorders-5-Text-Revision (DSM-5-TR) does not officially recognize CSBD as a diagnosis, employing terms such as ‘narrowly excluded’ to describe the omission of excessive sexual behavior disorders in the DSM-5 (1). Additionally, Sassover et al. argue that more attention should be paid to compulsive components in the manifestations of CSBD (13). However, symptomatic similarities between CSBD and other disorders in this category, such as OCD, body dysmorphic disorder, olfactory reference syndrome, hypochondriasis, and body-focused repetitive behavior disorder, are minimal. Among these disorders, avoiding negative states such as tension or fear is the focus, along with a lack of pleasant experiences or positive rewards. Given this, CSBD appears to be unsuitable for categorization under OCD. The current classification places CSBD alongside other impulse control disorders such as pyromania, kleptomania, and intermittent explosive disorders. However, the differences between the non-pathological and pathological states in these disorders are essential rather than frequency-based. Notably, CSB and all substance-related or behavior-related addictions, such as drinking, smoking, gambling, and gaming, are common behaviors in society.

The scientific community continues to gather knowledge regarding the nature of human sexual behavior. Three main models of sexual addiction have been proposed. They are based on impulse control disorders, OCD, and addictive disorders. The first model, proposed by Barth et al., is based on the compulsive-impulsive model, which is characterized by a lack of resistance to impulsive or harmful behaviors (55). This model interprets excessive sexual behavior as the inability to resist the impulse of sexual activities, which is in line with the DSM definition of impulse control disorder (56). Grant et al. investigated the prevalence of impulse control disorders among 204 psychiatric inpatients and found that 31% met the impulse control disorder criteria and 4.9% exhibited excessive sexual behavior (57). However, ICD-11 conceptualizes hypersexuality as an impulse control disorder but names it a CSBD, which puzzles many researchers and clinicians.

The second model introduces the term CSB, indicating a parallel relationship between OCD and sexual addiction, especially in terms of invasive and uncontrollable thoughts and behaviors. Sometimes, anxiety symptoms or psychological tension associated with the invasive thoughts that define OCD are observed in sexual addiction. Black et al. reported that among 36 patients with sexual addiction (28 males and 8 females), 42% reported repetitive and invasive sexual fantasies, and 67% reported poor self-esteem after sexual behavior (21). In another study, Raymond et al. reported that among 25 patients with sexual addiction (23 males and 2 females), 83% reported a decrease in mental tension after engaging in sexual behavior, and 70% described a sense of satisfaction after engaging in sexual behavior (22).

In the third model, hypersexuality is conceptualized as an addictive disorder (58). Craving starts as an early state, followed by repetitive behaviors that provide short-term pleasure or relief from mental distress. These behaviors become uncontrollable despite negative consequences. In a study by Potenza et al., 98% of patients reported withdrawal symptoms when sexual behavior was reduced, 94% reported difficulty or even an inability to control these behaviors, 92% reported spending excessive time on these behaviors, and 85% continued addictive behaviors despite negative physical or psychological consequences (59). The high prevalence of addictive comorbidities further supports this hypothesis: alcohol and psychotropic drugs (42%), gambling (5%), work (28%), shopping (26%), and eating disorders (38%) (2). These comorbidities may have pathophysiological bases similar to those of CSB.

The interaction between CSBD and neurochemical drugs suggests a correlation between CSBD and addiction. Naltrexone, an opioid antagonist mainly used to control alcohol or opioid dependence, has been found to be effective in reducing the impulses and behaviors related to CSBD, which is consistent with its role in treating gambling disorder (60). A literature review by Nakum et al. reported that the prevalence of CSBD is higher in Parkinson’s disease patients receiving dopamine replacement therapy, especially dopamine agonists (61). Kraus et al. believe that the association between dopamine replacement therapy and CSBD can address this classification issue (15). However, this evidence is inconclusive, as it has been found that dopamine replacement therapy can also cause various impulse control problems that do not necessarily lead to addictive behaviors (62, 63).

Both identifying CSBD as an impulse control disorder or addictive behavior face obvious limitations. Brand et al. highlighted the risk of excessive pathologization of sexual behaviors and emphasized the importance of differentiating between frequent and addictive behavior patterns (51). Addiction, impulse control, and other clinical paradigms carry the risk of oversimplifying complex clinical phenomena. Applying treatment models designed for other disorders to CSBD without careful planning and outcome studies is risky and irresponsible. For instance, using treatment models designed for alcohol or drug use disorders, which focus on moderation, for CSB is particularly concerning.

The definition and classification of CSBD within mental disorder taxonomies may represent a compromise among different conceptualizations, including impulse control disorder, OCD, non-paraphilic hypersexual disorder, behavioral addiction, and sexual disorder. Given the heterogeneity in CSBD’s clinical presentation and the uncertainty surrounding the accuracy of epidemiological, psychological, and neurophysiological research on CSBD, debates about its etiology and diagnostic classification are likely to continue. Recent studies have effectively outlined the current diagnostic guidelines and controversies surrounding CSBD, suggesting that further research is required to better understand the etiology, clinical manifestations, comorbid factors, and appropriate classification of CSBD (13, 51, 64).

In conclusion, there is currently insufficient scientific evidence to determine the most appropriate classification and symptomatology of CSBD (13, 64). Whether problematic sexual behavior is described as CSB, sexual addiction, or sexual impulse disorder, thousands of psychiatrists and psychotherapists worldwide are engaged in the diagnosis and treatment of such disorders. The psychiatric community and the entire medical field should prioritize accumulating and summarizing evidence-based medical evidence for the treatment of CSBD.

## 7 The diagnosis of CSBD

The diagnosis of CSBD requires a thorough assessment, including a comprehensive examination of sexual history and current symptoms. It also involves the assessment of somatic and psychiatric medical histories to rule out other potential causes, such as neurological disorders or medication side effects. Moreover, examiners must conduct a psychiatric assessment to identify comorbid mental health conditions, including mood and anxiety disorders, SUDs, post-traumatic stress disorder, and personality disorders.

From a diagnostic perspective, CSBD should be regarded as a distinct and well-defined clinical disorder differentiated from other disorders (such as paraphilias and persistent genital arousal disorder). It must be acknowledged that the sense of loss of control and the perception of the consequences of uncontrolled sexual behavior represent a subjective experience influenced by factors such as personal values, beliefs, cultural norms, environmental expectations, and personality traits. To avoid overpathologizing individuals with high-frequency sexual behaviors that are personally or socially unacceptable, a limitation has been added to the diagnostic criteria of CSBD; that is, an individual’s distress should not be solely related to moral judgment or social disapproval. Cultural norms significantly influence perceptions of “loss of control” and “abnormal sexual behavior,” particularly in non-Western societies. For example, stigma around premarital sex, same-sex relationships, or masturbation in collectivist cultures may lead to misattribution of culturally disapproved behaviors as pathological (18, 65). A cross-cultural study involving 42 countries found that CSBD prevalence varied from 2.1% to 8.9%, partly due to cultural differences in defining “distress” and “impairment” (18). In some contexts, individuals may report “loss of control” based on cultural guilt rather than clinical dysfunction, risking overpathologization. It is important to distinguish between individuals with high sex drive and those with CSBD. If a high sex drive does not cause distress or if the distress is only mediated by negative social norms (e.g., cultural restrictions and religious thoughts regarding sex and sexual desire), then individuals with a high sex drive should not be pathologized (11, 66).

Various questionnaires and interviews can assess CSB and help determine whether an individual should be diagnosed with CSBD. A comprehensive evaluation should include both a self-rating tool (e.g., Hypersexual Behavior Inventory-19, HBI-19) and standardized external ratings (e.g., CD-11). For future research and clinical studies, we recommend using CSBD-19 and CSBD-7 based on ICD-11, as these tools have demonstrated their effectiveness in 42 countries (18). According to the DSM-5 criteria, the Hypersexual Disorder Screening Inventory (HDSI), consisting of seven items, was developed for screening CSBD (67). The first five items assess the intensity of sexual fantasies, sexual urges, and sexual behaviors, and the last two items assess personal distress or social impairment. It can be administered as an interview or a self-report scale. Compared with other existing diagnostic tools, the HDSI is noted for its strong psychometric properties (68). If there are concerns about the risk of sexual crimes related to CSB, standardized risk assessment tools (such as STABLE-2007 or the Violence Risk Scale-Sexual Offender Version) can be used to evaluate this aspect (69, 70). Conducting high-quality, internationally standardized assessments of CSBD will help identify patients with CSBDs across different populations and will ultimately facilitate research on evidence-based and culturally sensitive prevention and intervention strategies (1, 65).

## 8 The treatment of CSBD

The main treatment goals for patients with CSBD involve enhancing sexual self-control; reducing problematic sexual behaviors; minimizing adverse consequences, lowering the risk of harm to oneself or others; and alleviating distress and impairment in personal, family, social, educational, vocational, or other significant functional domains (71). Discrimination, stigmatization, and morally inconsistent therapeutic interventions should be strongly opposed and avoided. This includes pathologizing the sexual behaviors of sexually diverse individuals, unilaterally prohibiting certain sexual behaviors (e.g., watching pornography and masturbation), applying addiction models to control sexual behaviors, and attempting to impose the moral or religious values of medical professionals under the guise of evidence-based treatment.

According to the current state of knowledge, CSBD arises from the complex interactions between biology, psychology, and culture. Therefore, clinicians need to understand and skillfully manage the relevant factors that contribute to and perpetuate excessive sexual behaviors. Initially, treatment should focus on achieving stability, motivation, and self-management. Later, the treatment should address the functions of the aforementioned sexual behaviors, intimate relationships, and relationship issues.

Depending on differences in patients, available resources, and specific needs, various psychotherapy models and techniques may be used. Individual therapy, group therapy, and couple therapy are possible treatment modalities. In a recent systematic review, Antons et al. summarized 24 intervention studies on CSBD (72). Among the reviewed studies, the most widely used components of cognitive-behavioral therapy were as follows: psychoeducation; motivation and the impetus for change (e.g., motivational interviewing); goal determination; awareness of thoughts, emotions, and beliefs; training in self-regulation and impulse management; skill training: development of problem-solving skills, conflict management, time management, and coping strategies; mindfulness and meditation practices; relapse prevention and maintenance planning; along with acceptance and commitment therapy. Many of these techniques are only applicable during the initial stages of treatment.

An expert group from the World Federation of Societies of Biological Psychiatry (WFSBP) recently proposed an algorithm for pharmacological treatment of CSBD (16). The extent of the intervention depends on the severity of the CSBD symptoms. The algorithm focuses on the evidence for the use of pharmacological treatments and classifies CSBD into three levels (mild, moderate, and severe) to guide the treatment plan. Typically, psychotherapy alone is used for first-level treatment and forms the basis for all subsequent treatments. In second-level treatment, the use of selective serotonin reuptake inhibitors or the opioid receptor antagonist naltrexone may be considered. In third-level treatment, a combination of these two drugs is recommended. While these drugs have shown some effectiveness in treating CSBD, they are “off-label” prescriptions, and no drug has been officially approved by regulatory agencies for the treatment of CSBD. In cases where CSBD coexists with paraphilic disorders, mainly considering the risk to others, drugs that reduce testosterone or gonadotropin-releasing hormone agonists may be used (73).

The summary of clinical research on the use of naltrexone is presented in **Table 4**. Naltrexone is a long-acting preferential opioid receptor antagonist that is widely used in the treatment of alcohol or opioid use disorders (74). Naltrexone may help reduce the potential for addiction by inhibiting endogenous opioids from triggering dopamine release in the nucleus accumbens (75). The gradual desensitization achieved through naltrexone may be associated with reduced pleasurable effects, helping individuals with CSB reduce and regain control over their sexual behavior.

**Table 4:** Studies of naltrexone in the treatment of CSBD.

Study subjects	Treatment conditions	Key outcomes	Side-effect profile	Tolerability
Double-blind study Lew-Starowicz et al. (79)	N = 73 heterosexual men with CSBD according to ICD-11. Group 1: Paroxetine 20 mg/d; Group 2: Naltrexone 50 mg/d; Group 3: Placebo.	Significant reductions in symptom severity were observed in all patients after 8 and 20 weeks, but there were no differences between the 3 groups. In clinical interviews, paroxetine and naltrexone were found to be more effective than placebo in reducing CSBD symptoms.	Sedation: 37.5% (most common, higher than placebo/paroxetine); Apathy: 8.3%; Weight gain: 4.2% (lowest among groups); No sexual dysfunction reported; No serious side effects reported	Discontinuation rate due to AEs: 12.5% (3/24 patients; primarily sedation); Adherence: No significant difference vs. other groups (p=0.78); Overall: Safe with acceptable tolerability, sedation notable but manageable
Open study Ryback (94)	N = 21 juvenile, legally-adjudicated sexually-offending patients. Naltrexone: initial dose 50 mg/d, mean dose 170 mg/d.	No significant efficacy was observed at doses less than 100 mg/day. At doses of 150–200 mg/day, 15 patients (71.4%) benefited. Efficacy was observed with 7.5 mg leuprolide per month in patients who did not respond to naltrexone (5 patients).	No changes in clinical chemistries (e.g., liver function, CBC); No reported significant systemic or organ-specific adverse effects.	Well-tolerated at doses of 100–200 mg/day; Dosages above 200 mg/day not more helpful; Symptoms recurred when dose tapered to 50 mg/day or discontinued, and resolved upon treatment resumption; No evidence of severe adverse reactions during treatment
Open study Raymond et al. (60)	N = 19 patients with paraphilic and non-paraphilic CSB. Naltrexone: 50–200 mg/d; 15 Patients concurrent use of SSRI or SNRI, two patients additionally bupropion, three patients additionally bupropion and SSRI.	Seventeen patients showed significant improvement in CSB symptoms. The mean effective dose was 104 mg/day and the mean duration of treatment for participants was 1 year.	Sedation (2 patients discontinued) Sleep interference + paresthesia (1 patient discontinued) Potential hepatotoxicity (FDA warning for doses >50 mg/d, but no LFT elevations in study when NSAIDs avoided)	89% (17/19) positive response (CGI 1–2) 26% (5/19) discontinued (3 for side effects, 2 for lack of efficacy) Most required doses >50 mg/d (mean 104 mg/d, max 200 mg/d) Well-tolerated with LFT monitoring and NSAID avoidance
Open study Savard et al. (95)	N = 20 men with CSBD according to ICD-11; 95% with excessive masturbation. Naltrexone: starting dose 25 mg, maintenance dose 50 mg/d for 4 weeks (1 case unchanged at 25 mg/d)	Naltrexone is feasible and tolerable and may reduce symptoms of CSBD.	Fatigue (55%); Nausea (30%); Vertigo (30%); Abdominal pain (30%); Apathy (15%); Headache (10%); Anxiety (10%); Sexual dysfunctions (10%); No serious adverse events reported	All 20 participants completed treatment; High adherence; minimal dose misses.; No serious adverse events; no liver function issues; Most side effects mild/transient; 3 had symptoms throughout; No treatment discontinuations due to side effects
Case studies: Grant et al. (96), Raymond et al. (97), Bostwick et al. (98), Kraus et al. (99), Camacho et al. (100)				

CSBD, compulsive sexual behavior disorder; CSB, compulsive sexual behavior; ICD-11, the 11th revision of the International Classification of Diseases; SSRIs, selective serotonin reuptake inhibitors; SNRIs, serotonin-norepinephrine reuptake inhibitors.

The summary of clinical research on the use of selective serotonin reuptake inhibitors is presented in **Table 5**. Many case reports and open studies have reported that selective serotonin reuptake inhibitors (SSRIs) are effective in treating paraphilic disorders and non-paraphilic CSB, although SSRIs are not officially indicated for these conditions (76). One mechanistic hypothesis links the anti-obsessive effects of SSRIs to the hypothesis that CSB are related to OCD and impulse control disorders. SSRIs may decrease sexual desire, weaken erectile function, and delay ejaculation by increasing the binding of serotonin to the 5-HT-2 receptors in the brain and spinal cord (77). Sexual dysfunction associated with the use of SSRIs has been reported during the treatment of mood disorders and may persist during longer treatment courses (78).

**Table 5:** Studies of SSRIs in the treatment of CSBD.

Study subjects	Treatment conditions	Key outcomes	Side-effect profile	Tolerability
Double-blind study Lew-Starowicz et al. (79)	N = 73 heterosexual men with CSBD according to ICD-11. Group 1: Paroxetine 20 mg/d; Group 2: Naltrexone 50 mg/d; Group 3: Placebo.	In clinical interviews, paroxetine and naltrexone were found to be more effective than placebo in reducing CSBD symptoms. Paroxetine reduced cravings for sexual contact and pornography, whereas the other two drugs did not.	Sedation: 29.2% (common, mild); Sexual dysfunction: Orgasmic: 2.8%; Erectile: 12.5%; Weight gain: 16.7%; Apathy: 8.3%; No serious side effects reported.	Discontinuation rate due to AEs: 8.3% (2/24 patients; sedation/sexual issues); Adherence: No significant difference vs. other groups (p=0.78); Overall: Well-tolerated; side effects mild/transient.
Double-blind study Wainberg et al. (101)	N = 28 homosexual men with CSBD. Group 1: Citalopram (20–60 mg/day); Group 2: Placebo; Treatment lasted 12 weeks.	After 12 weeks, CSB symptoms were reduced in both groups. The citalopram group showed greater decreases in libido, frequency of masturbation, and hours of pornographic image use per week.	Delayed ejaculation: Higher in citalopram vs. placebo; Mediated effects on masturbation/pornography use; Mild sexual side effects (SSE): No severe dysfunction or dissatisfaction reported	Completion rate: 85% (2 dropouts in citalopram group, non-SSE-related); Adherence: 98% appointment attendance Blood levels confirmed compliance (citalopram detectable, placebo undetectable) Safety: No serious adverse events; stable sexual satisfaction
Open study Kafka (102)	N = 10 men with nonparaphilic sexual addictions. Total 12 weeks: 6 patients fluoxetine 20–60 mg/day, 1 patient promethazine 225 mg/day, 1 patient lithium 1500 mg/day, 1 patient promethazine 125 mg/day þ lithium 600 mg/day, 1 patient fluoxetine 60 mg/day + trazodone 150 mg/day.	All but one patient treated with fluoxetine monotherapy showed significant improvement in symptoms.	No severe dysfunction or dissatisfaction reported	No serious adverse events; stable sexual satisfaction
Open study Kafka et al. (76)	N = 10 men with paraphilic disorder and non-paraphilic sexual addictions; N = 10 men with non-paraphilic sexual addictions. Fluoxetine: Starting dose 20 mg/day, maximum dose 60 mg/day, mean dose at 12 weeks 39.37 mg/day.	Significant reductions were observed in total sexual outlet, masturbation frequency, frequency of sexual activities, intensity of sexual desire after 12 weeks of treatment; Greater decreases were observed in the paraphilic group in total sexual outlet.	No severe dysfunction or dissatisfaction reported	4 men dropped out during the study (1 paraphilic and 3 non-paraphilic); Overall average reduction of total sexual outlet was 65.2%.
Open study Stein et al. (103)	N = 5 men with paraphilic disorder; N = 5 with non-paraphilic sexual addictions; N = 3 men with sexual thoughts or rituals. Fluoxetine up to 80 mg/d; Clomipramine up to 400 mg/d; Fluvoxamine up to 300 mg/d; Fenfluramine up to 40 mg/d.	There was no change in paraphilic fantasies or behaviors; non-sexual OCD symptoms decreased in 2 of 5 patients; and sexual OCD symptoms improved in 2 of 5 patients.	No severe dysfunction or dissatisfaction reported	No serious adverse events; stable sexual satisfaction; Only in two of the paraphilic patients did SSRI treatment led to a reduction in CSB, mainly decreased masturbation. A similar proportion was found for the CSBD patients with three non-responders within this group.
Open study Kafka (104)	N = 13 men with paraphilias; N = 11 men with paraphilia-related disorders. Sertraline mean dose 99.0 mg (range 25–250 mg/day) for 4–64 weeks.	Nine men who did not respond to sertraline were subsequently treated with fluoxetine. Of the 24 men who received sertraline and/or fluoxetine medication for at least 4 weeks, 17 (70.8%) achieved a clinically significant response.	Gastrointestinal distress (n = 3); Sexual dysfunction (n = 3); Fatigue (n = 2); Increased depression (n = 2); Headache (n = 1)	Low dropout rate: 24/26 men completed ≥4 weeks of treatment.; Mean treatment duration: 17.4 ± 18.6 weeks (range: 4–64 weeks)
Case studies: Elmore (105), Gola et al. (106)				

SSRIs, selective serotonin reuptake inhibitors; CSBD, compulsive sexual behavior disorder; ICD-11, the 11th revision of the International Classification of Diseases; CSB, compulsive sexual behavior; OCD, impulse control disorders.

In 2022, a placebo-controlled, double-blind RCT compared the tolerability and effectiveness of paroxetine and naltrexone in the treatment of CSBD (79). This study, involving 73 heterosexual men (average age 35.7) diagnosed with CSBD by ICD-11 criteria, compared naltrexone (50 mg daily), paroxetine (20 mg daily), and placebo over 20 weeks. The frequency of sexual urge episodes self-reported by patients at week 20 was reduced. In clinical interviews, paroxetine and naltrexone were found to be more effective than the placebo at reducing CSBD symptoms after 8 and 20 weeks, respectively.

Overall, there is currently very little high-level evidence regarding the pharmacological treatment of CSBD, and most studies are case reports. Notably, CSBD treatment plans have largely overlooked the specificity of sexual symptoms, moral incongruence, pornographic conflicts, and previous trauma. Additionally, they have neglected to address issues related to attachment, intimate relationships, and existing sexual function problems.

## 9 Limitations and future directions

### 9.1 Prospects for etiology research

There is currently insufficient scientific evidence to definitively categorize and symptomatically classify CSBD. The lack of etiological understanding has led to interventions based on flawed reasoning, which may result in erroneous, iatrogenic, and misleading outcomes. It is a misconception to believe that medications, such as naltrexone or SSRIs, can address all issues related to CSBD. In many cases, patients have experienced worsening conditions, intolerable side effects, or merely a placebo effect from the drugs (80). While viewing CSB as an addiction or impulse control disorder might seem like a step backward, future research should focus on exploring the underlying causes of CSBD, developing interventions that target these causes, and assigning research participants to optimal treatment conditions based on these etiological assessments.

### 9.2 Evaluation of treatment efficacy

Most reports on CSBD treatments are case reports or series, and studies on treatment efficacy often have methodological biases. Additionally, previous research often relied on self-reported sexual activity to evaluate treatment outcomes, focusing only on subjective improvements without standardized and reliable methods for measuring sexual behavior. More prospective studies are needed, using various measurement tools before and after interventions, including structured psychometric assessments, behavioral evaluations, and neuroimaging. National or international collaborative research, including large-scale CSBD cohort studies with long-term follow-up, is necessary to confirm credible data on the effectiveness of CSBD treatments.

### 9.3 Addressing polyaddiction

Polyaddiction is a critical aspect often overlooked in CSBD treatment. It is common for CSBD to co-occur with various other impulsive and addictive behaviors, such as substance use, problematic gaming, and gambling, as well as paraphilic disorders. This overlap is referred to as addiction interaction disorder in the sexual addiction community. For example, common addiction interaction disorder involves a combination of cocaine abuse, alcohol misuse, and excessive sexual behavior (81). Due to the shame associated with CSBD, patients often do not discuss it openly. In most substance abuse treatment centers, individuals seeking treatment for these behavioral combinations may have their sexual issues overlooked and receive treatment only for substance abuse and alcohol dependency. Unresolved sexual addiction can sometimes be a relapse trigger for substance and behavioral addictions. These issues underscore the need to assess CSBD within addiction treatment settings, especially where behavioral addiction is already the focus of treatment. Moreover, comprehensive education and interventions for patients with polyaddiction are crucial, with measures taken to reduce sexually transmitted infections, prevent human immunodeficiency virus, decrease alcohol/illicit drug intoxication and related adverse effects, and lower the risk of non-consensual sexual activities and sexual violence.

### 9.4 Addressing sexual dysfunction

Patients with CSBD often exhibit poor sexual performance. Individuals with CSBD display higher levels of sexual anxiety, sexual depression, external sexual control, and fear of sexual relationships than does the general population. Moreover, the severity of CSBD is inversely correlated with sexual self-esteem, internal sexual control, sexual awareness, sexual confidence, and sexual satisfaction (82). Individuals with sexual addiction may vigorously pursue their dysfunctional sexual behaviors, but they often face various forms of sexual dysfunction (83). Premature ejaculation, erectile dysfunction, impaired desire, sexual aversion, sexual anorexia, and anorgasmia are common among individuals with sexual addiction, particularly those in long-term partnerships or stable relationships. Consequently, integrated treatment is often necessary to foster healthy sexual relationships and satisfy sexual experiences during recovery. Sexual function therapy is typically initiated after achieving initial control of the behavioral disorder. Continuous monitoring and assessment of a patient’s sexual behavior are essential, with prescriptions tailored to address primary, secondary, and/or situational sexual dysfunctions.

### 9.5 Diagnosing and treating ADHD-CSBD comorbidity

ADHD, a neurodevelopmental disorder characterized by core symptoms of inattention, hyperactivity, and impulsivity, is often accompanied by early-onset emotional dysregulation, oppositional behaviors, or disorganization (84). The association between CSBD and ADHD reflects a complex interplay of shared and distinct mechanisms. Both disorders are characterized by deficits in self-regulation, particularly in impulse control and emotional regulation, which may underlie their comorbidity (85).

In clinical samples, ADHD symptoms are prevalent in individuals with CSBD, with historical studies reporting ADHD rates of 20–27% in men with CSB, often manifesting as the inattentive subtype (32, 86). According to a prior study, the co-occurrence rate of CSB in individuals with ADHD ranges between 5% and 12%, depending on the diagnostic threshold applied for ADHD (87). In a German online study involving 139 adults with ADHD (n = 89 women), approximately one-quarter (24.5%) reported CSB, with notable gender differences: 14.6% of women and 45.5% of men were affected. Despite these elevated co-occurrence rates, the study found no significant difference in the prevalence of CSB between adults with ADHD and those without (84).

A key diagnostic challenge is avoiding “overshadowing”, where impulsive sexual behaviors in ADHD might be misattributed to CSBD or vice versa. Both disorders involve difficulties with impulse control, but CSBD is characterized by recurrent, distressing sexual urges that persist despite negative consequences, whereas ADHD primarily involves sustained deficits in attention and hyperactivity (88). Clinicians must conduct thorough assessments to distinguish whether sexual compulsivity represents a primary disorder or secondary symptom of ADHD, particularly in cases where substance use or comorbid mood/anxiety disorders complicate presentation.

In terms of interventions, pharmacological approaches include psychostimulants, which may reduce both ADHD symptoms and sexual compulsivity by enhancing prefrontal control—though evidence is mixed, as seen in Kafka and Hennen’s positive findings versus Bijlenga et al.’s null results—thus necessitating individualized care (87, 89). Adjunctive medications like SSRIs, used for CSBD, help regulate mood and impulse control in comorbid cases but have limited impact on ADHD symptoms. Dual-diagnosis care is crucial, as untreated ADHD can hinder CSBD treatment adherence and vice versa, necessitating collaboration between sexologists and ADHD specialists (85). Such collaboration should involve integrating standardized assessments and longitudinal monitoring to tailor interventions effectively.

In summary, the complex interplay between CSBD and ADHD requires clinicians to avoid diagnostic oversights, recognize shared and distinct traits, and adopt integrated treatment strategies that combine pharmacological, behavioral, and collaborative approaches.

### 9.6 Focus on women and sexually diverse populations

The current understanding of CSBD is predominantly based on research on heterosexual male samples from Western countries. Little is known about the characteristics of CSBD among populations in non-Western cultures, as well as among women and sexually diverse groups. This indicates a methodological oversight where potential sex and cultural differences are frequently neglected. In fact, existing literature has reported differences in the prevalence and types of CSB when accounting for gender and sexual orientation. In a clinical study involving men (n = 64) and women (n = 16) with self-identified CSB, the most common problematic sexual behavior among men was pornography use (reported in 82% of cases), compared to 50% of women. Conversely, the most frequently cited behavior among women was engaging in sexual activity with consenting adults (88%), versus 36% of men (90).

A population-based study (n = 18,034) further indicated that non-heterosexual men—and to a lesser degree, non-heterosexual women (e.g., gay, bisexual individuals)—along with transgender and queer persons exhibited the highest prevalence of CSB indicators (e.g., masturbation frequency, number of sexual partners, pornography consumption frequency). This group also recorded the highest scores on the HBI-19 (91). Notably, however, these findings contrast with other research (92). For instance, Gleason et al. estimated that 7.9% of U.S. gay men exhibit clinically significant CSB—a prevalence not statistically higher than that observed in the general population (93).

These differences can affect risk factors, the severity of symptoms, the accuracy of diagnosis, and the effectiveness of treatment options for CSBD. More research is required on the prevalence, incidence, etiology, diagnostic criteria, comorbidities, sexual behavior patterns, and barriers to seeking help among women, impoverished and racial/ethnic minority groups, homosexuals, bisexuals, transgender individuals, people with physical and intellectual disabilities, and people from diverse cultural backgrounds. Bridging this research gap is crucial for improving clinical care, developing targeted interventions, and enhancing awareness of CSBD among the public, healthcare providers, and policymakers.

### 9.7 Enhancing training in sexual medicine

Given the sexual origins and consequences of CSBD, as well as the need for evaluation, diagnosis, and treatment of a range of sexual behaviors and disorders within a sociocultural context, expertise in clinical sexology or sexual medicine is required. Clinicians need to adopt a non-judgmental, positive attitude, understand sexual diversity, and identify the individual mechanisms that lead to uncontrolled sexual behavior, associated distress, and negative outcomes. The diagnostic process should also involve a detailed, thorough, and unbiased inquiry into partner-related issues and the patient’s sexual history. Treatment should focus not only on restricting sexual behaviors and preventing negative outcomes, but also on developing or restoring normal sexual and partner relationships. Therefore, clinicians need to knowledgeable in sexual medicine, including the assessment and management of various medical conditions and the interplay between various medications, sexual function, and behavior. Considering the limited focus on CSBD in current medical education, healthcare professionals treating CSBD may have limited knowledge of certain aspects of sexology. This highlights the need for interdisciplinary consultations and training programs.

## 10 Conclusions

CSBD is not a novel phenomenon or diagnostic entity but a problem described since the inception of the psychiatric-psychotherapeutic and sexual sciences. Despite being classified as an impulse control disorder, the precise etiological categorization of CSBD remains controversial. The diagnosis and treatment of patients with CSBD should integrate biological, psychological, and social factors with expertise in sexual medicine by employing a comprehensive and holistic therapeutic approach. This treatment aims not only to control abnormal sexual desires and behaviors but also to assist patients in achieving a healthy and satisfying sexual life and well-being.
